# Microstructure, Mechanical Properties and Additive Manufacturing of Steels

**DOI:** 10.3390/ma19102140

**Published:** 2026-05-20

**Authors:** Róbert Bidulský, Jana Bidulská

**Affiliations:** 1 Bodva Industry and Innovation Cluster, Budulov 174, 04501 Moldava nad Bodvou, Slovakia; 2 Department of Plastic Deformation and Simulation Processes, Institute of Materials and Quality Engineering, Faculty of Materials, Metallurgy and Recycling, Technical University of Kosice, Park Komenského 11, 04200 Košice, Slovakia; jana.bidulska@tuke.sk

## 1. Introduction and Scope

Recent developments in the additive manufacturing (AM) of steels focus on microstructural engineering to overcome traditional trade-offs between strength and ductility, often utilising high-speed machine learning or mathematical simulation for alloy discovery and hybrid manufacturing techniques to optimise performance. The topic of microstructure and mechanical properties always targets a material’s properties. It is therefore useful to focus on the parameters that link properties and microstructure [1,2,3,4,5,6,7,8,9,10,11] and on how to drive them using simulation and modelling [12,13,14,15,16,17,18,19,20], including joining, metal forming and AM techniques. Advanced AM steels are now reaching or exceeding the performance of their conventionally manufactured (CM) counterparts through specific strengthening mechanisms [1,21,22,23].

## 2. An Overview of the Microstructure, Mechanical Properties and Additive Manufacturing of Steels

The Special Issue on the Microstructure, Mechanical Properties, and Additive Manufacturing (AM) of Steels contributes to developments in the field of AM [24,25,26,27,28]. Reading the collected papers, some conclusions can be drawn.

The paper [24] examines the microstructural characteristics and corrosion resistance of super duplex stainless steel (SDSS) produced via Laser Powder Bed Fusion (LPBF). It utilises X-ray diffraction (XRD), microstructural analysis (SEM), potentiodynamic polarisation tests and electrochemical impedance spectroscopy (EIS). The results established that solution annealing and stress relieving at 400 °C improve resistance, while treatment at 550 °C reduces it due to the precipitation of chromium nitrides. The authors also explain the physical nature of the changes (e.g., the effect of dislocation density on XRD peak width or the impact of nitrides on the passive layer).

The study by Pereira et al. [25] focuses on the design and analysis of eco-friendly, fluorine-free mould fluxes intended for the continuous casting of peritectic steels, aiming to replace toxic CaF_2_ with alternative oxides such as Na_2_O, TiO_2_ and B_2_O_3_ while maintaining essential thermophysical properties. To achieve this, the authors employed a comprehensive methodology combining a 2^4^ factorial design with thermodynamic modelling via FactSage 8.3, complemented by experimental verification through viscosity measurements, differential scanning calorimetry (DSC) for crystallisation kinetics, and phase identification using X-ray diffraction (XRD) and scanning electron microscopy (SEM-EDS). The research findings demonstrated that a specific fluorine-free formulation (Sample A) exhibits intense precipitation of merwinite and perovskite, effectively replacing cuspidine in controlling horizontal heat transfer. Furthermore, the study proves that an optimised combination of Na_2_O and TiO_2_ yields a balanced viscosity and crystallisation behaviour comparable to traditional fluorine-based fluxes. This work represents a highly relevant advancement for sustainable “Green Steelmaking” by eliminating hazardous emissions and equipment corrosion while addressing the specific technological risks associated with the non-uniform solidification of peritectic steels in industrial practice.

The study by Sang et al. [26] investigates the effects of plate thicknesses (2 mm, 3 mm and 4 mm) on the microstructure and mechanical properties of laser-welded 22MnB5 hot-forming steel, aiming to expand its applications from thin automotive components to heavy-duty machinery and the military sector. The researchers employed a comprehensive experimental methodology that involved direct hot-forming of the base metal to a martensitic state, followed by fibre-laser butt-welding and advanced characterisation through tensile testing, Vickers microhardness measurements, and high-resolution imaging techniques such as SEM, TEM and EBSD. The results revealed that as plate thickness increased from 2 mm to 4 mm, the tensile strength of the welded joints decreased from 1489 MPa to 1275 MPa, representing a drop from 96% to 88% of the base metal strength. This reduction in mechanical performance is attributed to the growth of martensite grains, a transition from fine acicular to larger island-like structures, and a significant decrease in dislocation density within the heat-affected zone, which was identified as the weakest region where fractures occurred. Despite these changes, the finding that all joint thicknesses maintained over 85% of the base metal’s strength confirms the strong industrial potential for laser welding thicker hot-forming steels. This research provides highly relevant and timely data for optimising high-power laser welding parameters for advanced structural applications.

The study by Mroziński et al. [27] analyses the impact of microstructural anisotropy on the low-cycle fatigue (LCF) of S420M structural steel, specifically comparing samples collected parallel and perpendicular to the rolling direction. The research methodology integrated static tensile testing (Z-tensile test), Rockwell hardness measurements across the plate cross-section, and low-cycle fatigue tests under both constant-amplitude and programmed loading conditions, supplemented by scanning electron microscopy (SEM) for microstructural and fracture surface characterisation. The results demonstrated that while the rolling direction has a negligible effect on basic static strength parameters such as tensile and yield strength, it exerts a profound influence on fatigue properties, with perpendicular samples exhibiting a dramatic reduction in fatigue life ranging from 50% to nearly 300% depending on the strain level. Furthermore, experimental verification of the Palmgren–Miner linear hypothesis confirmed that using fatigue data from longitudinal samples to calculate the life of components loaded in the transverse direction leads to dangerously overestimated results. This research is highly relevant because it addresses a critical safety gap in current engineering standards, demonstrating that meeting static requirements for through-thickness properties does not guarantee adequate fatigue resistance in heavy-duty welded structural nodes.

The study by Widomski et al. [28] presents a comparative analysis of five distinct additive manufacturing technologies—Fused Deposition Modelling and Sintering (FDMS) via Desktop Metal and Zetamix systems, Binder Jetting (BJ), Laser Powder Bed Fusion (LPBF), and Directed Energy Deposition (DED)—applied to H13 tool steel, which is essential for hot-work tooling applications. The research focuses on evaluating microstructure, porosity levels and the material’s response to post-processing heat treatments such as quenching and tempering. The experimental methodology involved utilising light optical microscopy, scanning electron microscopy with energy-dispersive spectroscopy, Vickers microhardness testing, and quantitative porosity analysis. The findings indicated that LPBF and DED achieved the highest densities with porosity levels below 0.1%, whereas sinter-based FDMS methods exhibited significantly higher porosity, ranging from 6.0% to 9.3%, thereby limiting their suitability for functional tooling. After specialised heat treatment, high-density samples (BJ, LPBF and DED) achieved satisfactory hardness levels between 600 and 700 HV0.5, with Binder Jetting specifically demonstrating a secondary hardening effect due to the precipitation of vanadium and chromium carbides during tempering at 550 °C. This research is highly relevant as it addresses a critical research gap by providing a direct comparison of diverse additive manufacturing methods under unified experimental conditions, offering vital insights for the development of advanced tools with conformal cooling channels.

## 3. Conclusions

The research published in the Special Issue Microstructure, Mechanical Properties, and AM of Steels confirms that additive manufacturing and advanced metallurgical processes are fundamentally transforming the design of steel components. In conclusion, the synergy among experimental characterisation, mathematical simulation and additive technologies enables the overcoming of traditional limits in metallurgy, paving the way for a new generation of high-performance and environmentally sustainable steel structures.
